# Cryoprotective effect of silver carp muscle hydrolysate on baker's yeast *Saccharomyces cerevisiae* and its underlying mechanism

**DOI:** 10.1002/fsn3.1290

**Published:** 2019-11-25

**Authors:** Faxiang Wang, Sijia Xiong, Xianghong Li, Jian Yu, Yiqun Huang, Yongle Liu

**Affiliations:** ^1^ Hunan Provincial Engineering Technology Research Center of Aquatic Food Resources Processing School of Chemistry and Food Engineering Changsha University of Science and Technology Changsha China

**Keywords:** cryoprotective effect, fish muscle hydrolysate, internal ice formation, ultrastructure, yeast

## Abstract

Cryoprotective effect of silver carp muscle hydrolysate (SCMH) on baker's yeast (Saccharomyces cerevisiae) was examined by analyzing the growth and survival of the yeast during freeze–thaw cycles, and the physicochemical properties [ultrastructure, intracellular proteins and fatty acids, external ice formation (EIF) and internal ice formation (IIF), freezable water content] of yeast cells with or without SCMH through transmission electron microscopy, SDS‐PAGE, GC‐MS, and differential scanning calorimetry. The 4% of SCMH treatment exhibited good yeast cryoprotective activity and increased the yeast survival rate from 0.71% to 90.95% after 1 freeze–thaw cycle as compared to the control. The results demonstrated that the addition of SCMH could attenuate the freeze damage of yeast cells, prevent the degradation or loss of soluble proteins, and increase the composition and absolute content of fatty acids. Besides, the addition of 4% SCMH caused a drop in the EIF peak temperature (from −17.95℃ to −25.14℃) and a decrease in the IIF and freezable water content of yeast cells.

## Graphical abstract

Silver carp muscle hydrolysate (SCMH) has good cryoprotective effect on baker's yeast* Saccharomyces cerevisiae*. The underlying mechanisms are that SCMH can attenuate proteins lost, increase expression of fatty acids in frozen yeast cells, increase the proportion of nonfreezable water, and decrease IIF in yeast cells to alleviate freezing injuries.

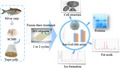

## 1 INTRODUCTION

The physical and chemical properties of frozen dough as affected by various factors have been extensively studied over the past four decades, which leads to a substantial improvement in the overall quality (such as textural properties and product consistency) of the resultant baked products and meanwhile contributes to an increase in the demand for frozen dough products, especially for individuals with fast‐paced lifestyle (Ding et al., 2015; Inoue, Sapirstein, Takayanagi, & Bushuk, 1994; Meric, Guilois, Neyreneuf, & Molard, 1995; Oda, Uno, & Ohta, 1986; Ribotta, León, & Añón, 2003). However, the negative effects of both freezing and frozen storage of dough on the quality of the subsequently baked products still could not be completely resolved, and the freezing damage to yeast cells was regarded as one of the main culprits contributing to the quality loss of frozen dough (Meric et al., 1995; Oda et al., 1986). Therefore, improving the freezing tolerance of yeast helps minimize the quality deterioration in bread prepared from frozen dough. This could be theoretically settled by isolation of freeze‐tolerant strains of yeast (Takagi et el., 1997), but the addition of a suitable cryoprotective additive has been considered as one of the most effective solutions (Hirasawa, Yokoigawa, Isobe, & Kawai, 2001; Hubálek, 2003; Yokoigawa, Sato, & Soda, 2006).

Since the discovery of the protective effects of glycerol for some microbial cells (including yeast) against freezing damage that marked the beginning of modern cryotechnology, more and more researches have been performed to screen and investigate the additives with cryoprotective effects for microorganisms used in the freezing process. Hubálek (2003) reviewed the protectants used in the cryopreservation of microorganisms and pointed out that ovalbumin, gelatin, casein hydrolysate, and blood sera from some animals showed good cryoprotective effects on yeast. In recent years, some novel antifreeze improvers, such as antifreeze proteins (AFPs) (Ding et al., 2015), antifreeze peptides (APPs) (Chen, Wu, Li, & Wang, 2017), extracellular ice nucleators (ECINs) (Shi, Yu, & Lee, 2013), and ice‐structuring proteins (ISPs) (Jia et al., 2012; Xu, Huang, Wang, & Patricia, 2009), have been utilized to preserve yeast cells from freezing injuries. These antifreeze improvers could minimize or prevent freeze damage to yeast cell, but their availabilities are limited or their production costs are not inconsiderable, which greatly restricts their application in frozen food industry.

Lately, researchers found that fish protein hydrolysates showed good antifreeze property in frozen food systems. For example, Mueller and Liceaga (2016) reported silver carp (*Hypophthalmicthys molitrix*) muscle hydrolysates imparted antifreeze property equal to or better than the commercial cryoprotectants through increasing the water holding capacity and the amount of unfrozen water in the muscle (mince) food system. Silver carp is a kind of widely cultured freshwater fish with low value in China. It would be a competitive way of value‐added utilization, if a yeast cryoprotectant could be derived from the hydrolysate of silver carp muscle. Fortunately, our previous research results (Xiong et al., 2018) have proved that the muscle hydrolysates derived from silver carp (SCMHs) were able to increase the survival ratio of yeast in suspensions and the incorporation of 4% (w/v) of SCMH prepared from an optimized hydrolysis process exhibited good yeast cryoprotective property. However, the underlying mechanism governing the cryoprotective effect of these SCMHs on yeast is still unknown and further investigations on this cryoprotective effect are much needed.

The purpose of this study was to further investigate the cryoprotective effects of SCMH on yeast cells undergoing freeze–thaw treatments and its underlying mechanism. The survival rates, changes in the structure and two primary components (protein and lipid) of yeast cells undergoing freeze–thaw treatments with and without the addition of SCMH were tested. In addition, the intra‐ and intercellular ice formation of frozen yeast suspension were evaluated.

## 2 MATERIALS AND METHODS

### 2.1 Materials

Silver carp (*Hypophthalmicthys molitrix*) of approx. 1,500 ± 50 g were purchased from a local vendor in Changsha, China. Protamex with declared activity of 1.5 AU/g was purchased from Novozymes Biotech Inc., Denmark. Baker' yeast (*Saccharomyces cerevisiae,* CICC 1481) used in this study was brought from China center of industrial culture collection (CICC). All culture medium, such as malt extract medium, was brought from Guangdong Huankai Microbial Sci. & Tech. Co., Ltd. All chemicals used in this study were at least of analytical grade.

### 2.2 SCMH Preparation

Live silver carp (*n* = 5) were briefly reared in ice water mixture for 1 hr, immediately killed and filleted(about 100 g per piece), rinsed with cold, deionized water, and then minced using a meat grinder and homogenized in a blender with deionized water (1:5 w/v) for about 2 min. Next, the slurry was transferred to an enzyme‐catalyzed reactor (BIOTECH‐5M; Shanghai Baoxing Bio‐Engineering Equipment Co. Ltd) and its pH was adjusted to 6.5. Protamex was added at 3% (w/w) of the weight of the fish muscle‐water mixture. During the enzymatic hydrolysis process, 1.0 mol/L NaOH solution was added automatically to stabilize the pH at 6.5 ± 0.05, the total volume of NaOH solution was recorded to calculate the degree of hydrolysis (DH) based upon a pH‐Stat method (Alarcón, Moyano, & Díaz, 2002). The enzymatic hydrolysis was carried out at 50°C for 30 min. Following this, the fish hydrolysate was pasteurized, centrifuged and the supernatant was freeze‐dried as the method described by Mueller and Liceaga (2016). The process was repeated for three times, and each time a different batch of live fish was collected from the same vendor. The DH of the fish hydrolysate was 13.57 ± 0.56.

### 2.3 Preparation of yeast culture

To prepare yeast culture, one loopful of culture from yeast slant was transferred aseptically to a 100 ml shake flask with 10 ml of malt extract medium and incubated on a rotary shaker operating at 160 r/min for 24 hr at 28°C. Next, 5 ml of this liquid culture was used as seed to inoculate into 50 ml of malt extract medium and grew at the same conditions. This 50 ml malt extract medium culture grown in a 250 ml shake flask was immediately used for the subsequent experiments.

### 2.4 Freeze–thaw treatment of yeast suspensions

To prepare yeast suspensions with SCMH (4% w/v), 0.4 g SCMH was suspended in 9.0 ml sterilized water and then rapidly mixed into 1.0 ml yeast broth culture. Meanwhile, a yeast suspension without SCMH added was also prepared. The suspensions were immediately treated with two freeze–thaw cycles. Each freeze–thaw cycle consisted of freezing at −20 ± 2°C for 18 hr and thawing at 4 ± 2°C for 6 hr. Yeast suspension with 8% (w/v) of glycerol was used as positive control.

### 2.5 Yeast survival rate and growth assays

The yeast survival ratio was defined as the percentage of the colony‐forming unit (CFU) of viable yeast cells before and after a freeze–thaw treatment. The CFU of yeast was measured via the standard plate count as follows. The dilutions of yeast suspension in sterilized water (10‐ to 10^5^‐fold gradient dilution) were spread on potato dextrose agar (PDA) plates and incubated for 48 hr at 28°C before the numbers of colonies were counted.

### 2.6 Transmission electron microscopy (TEM) analysis

Yeast suspensions (1.0 ml, with 0 or 4% SCMH) subjected to 0 or 1 freeze/thaw cycle were centrifuged at 12,000 × g, 4°C for 15 min (Eppendorf 5430R, Hamburg, Germany). The pellets were collected and followed the fixation/embedding protocol as described by Thimon, Peypoux, Wallach, and Michel (1995) with minor modifications for TEM analysis. In brief, yeast pellets were firstly fixed using 2.5% (w/v) glutaraldehyde solution, washed with 0.1 mol/L phosphate buffer (pH 7.4), postfixed with 1.0% OsO_4_ solution for 2 hr at 4°C and washed three times with fresh buffer as above, then dehydrated with a series of acetone solutions of increasing concentration, beginning with 50% and progressing through 50%, 70%, 90%, and 100% absolute acetone. These samples were infiltrated in a 1:1 mixture of acetone and Poly Bed 812 resin (Polysciences, Inc.) for 12 hr at 37°C, embedded in Poly Bed 812 resin overnight, and then polymerized at 60°C for 24 hr. Next, the embedded samples were ultrathin‐sectioned using a microtome equipped with a glass knife and doubly electron‐strained using 3% uranyl acetate for 12 min and lead nitrate for 5 min. Finally, the thin sections of 50–100 nm were observed with a TEM (Hitachi HT7700 TEM, Hitachi Ltd.).

### 2.7 Protein extraction and SDS‐PAGE analysis

An SDS‐PAGE method (Laemmli, 1970) was employed to assess whether the addition of SCMH affected the protein composition in yeast cells undergoing a freeze–thaw treatment. In brief, one gram of yeast pellet with or without SCMH described was added in 1.0 ml of 2 × Laemmli loading buffer containing 0.5 mmol/L PMSF (phenylmethanesulfonyl fluoride) and homogenized for 5 min, incubated at room temperature for 1.0 hr, heated in boiling water bath for 3 min, and centrifuged (14,000 g) for 15 min. The supernatants were subjected to a SDS–10% polyacrylamide gel electrophoresis (SDS‐PAGE), and proteins were visualized by staining with Coomassie Brilliant Blue R‐250.

### 2.8 Fatty acids extraction and GC‐MS analysis

The fatty acids composition of yeast cells were analyzed based on a modified GC‐MS method involving the methylation of fatty acids (Peršurića, Saftića, Mašekb, & Pavelić, 2018). In brief, 1.0 g of yeast pellet was suspended in 4.0 ml methanol and ultra‐sonicated for 60 min (XO‐SM200, Nanjing Xianou Instruments Manufacture Co., Ltd.) at each 1 s work time and 1 s rest interval, and then stored at −20℃ for 30 min. This procedure was repeated three times. Then, 100 μl cell suspension was taken out and 1.6 ml of methanol, 0.4 ml of hexane, and 200 μl of acetyl chloride were added. The well‐mixed sample was held at 80 ℃ in a water bath for 60 min and cooled down to room temperature. Next, the sample was added with 5.0 ml of 6% (m/v) Na_2_CO_3_ solution, centrifuged for 5 min (5,000 g) and the hexane layer in supernatant was collected. Following this, 0.5 ml of hexane was added again for re‐extraction with the same condition. The hexane layers were combined and freeze‐dried, redissolved in 200 μL and centrifuged for 10 min (15,000 g). Finally, 1.0 μl supernatant was taken out for GC‐MS analysis using a triple quadrupole GC‐MS (GCMS‐TQ8050 NX, Hitachi Ltd) equipped with a Rxi®‐5Sil MS column (30 m, 0.32 mm ID, 0.25 μm). The column temperature was increased from 80°C to 300°C at the rate 5°C/min, then held for 2 min at 300°C. The pressure of carrier gas (helium) was 18.9 KPa, and the column flow was 1.40 ml/min. Spectra were recorded in full scan mode from 30 m/z to 800 m/z with 0.3 s/scan. Peaks generated by GC‐MS were identified by matching the mass spectra and confirmed by comparing with a reference mixture of 37 fatty acids methyl esters (FAME) Mix (AccuStandard, Inc). Absolute amounts of individual fatty acids were calculated by using both an external standard and the moisture content of the sample. The total cellular content of fatty acids (% of dry weight) was defined as the sum of absolute amount of all individual fatty acids.

### 2.9 Determination of freezable water content

The freezable water content in yeast cells after a freeze–thaw treatment was measured using differential scanning calorimetry (DSC) as follows (Zhang, Zhang, Wang, & Guo, 2008). Accurately weighed 5.0 mg of yeast pellet was transferred to a DSC sample pan, sealed, and stored at −30°C for 48 hr. After that, the sealed pan was quickly moved to a DSC instrument (DSC Q2000, TA Instruments). Each sample was held at −30°C for 5 min and then heated to 15°C at the rate of 3°C/min. The endothermic enthalpy (Δ*H*_W_, J/g) of the yeast cells was recorded. The freezable water content (φ) of yeast cells is expressed as:

(1) $$\phi =\frac{\Delta H_{W}}{\Delta fusH_{W}\times W_{A}}\times 100\%$$

where Δ_fus_ *H*_W_ is the endothermic enthalpy of water(Δ_fus_ *H*_W_ = 333.31 J/g), *W*_A_ is the moisture content of yeast cells before the freeze–thaw experiment and determined by drying the samples to constant weight at 105°C.

### 2.10 Analysis of external and internal ice formation

External ice formation (EIF) and internal ice formation (IIF) of yeast cells were analyzed according to the method reported by Jia et al. (2012). In brief, 0 or 0.4 g SCMH was suspended in 10.0 ml yeast broth culture and mixed for 5 min at 4°C, using as the control for yeast suspension (CTRL) or experimental group (4% of SCMH), respectively. Then, 5 μl yeast suspension was transferred to a differential scanning calorimeter (DSC) sample pan, and the exothermic curve of yeast cells in the process of cooling was measured using a DSC (DSC Q2000; TA Instruments). Each sample was cooled from an initial temperature of about 10°C to a target temperature of −40°C at a cooling rate of 10°C/min and then held at −40°C for 5 min.

### 2.11 Data analysis

Three replicates or more were analyzed for all experiments. Data were analyzed by analysis of standard deviations and variances using SPSS statistical package (Windows version 19.0). For the normally data of survival ratio and fatty acid's absolute content, Duncan's multiple range test (DMRT) was used to determine statistical significance (*p* < .05).

## 3 RESULTS

### 3.1 Effect of SCMH on the survival rate of yeast

**Figure 1:**
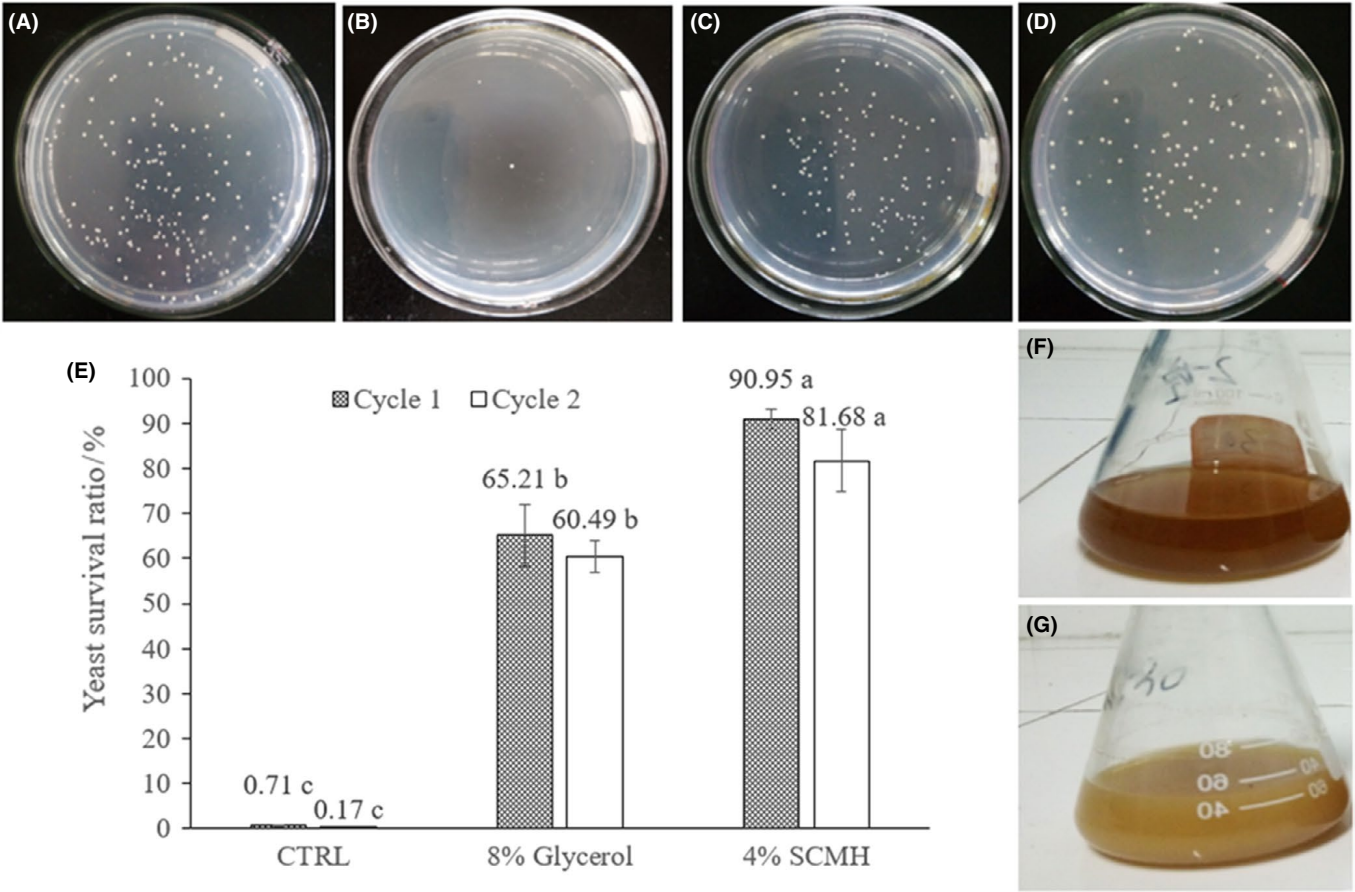
Colony growth (10^–5^) of yeast in petri dish before (A) and after one cycle of freeze‐thaw treatment with the addition of 0% of SCMH (B), 4% of SCMH (C), and 8% of glycerol (D); as well as the survival rate of yeast after one and two cycles of freeze–thaw treatment with and without (CTRL) the incorporation of SCMH or glycerol (E). Values bearing different lowercase letters (a, b, c) above each bar were significantly different (*p* < .05). Rejuvenation of yeast in triangular flask using freeze–thaw‐treated yeast suspensions with 0% (F, CTRL) and 4% (G) of SCMH

The effects of SCMH on the growth and survival rate of yeast after freeze–thaw treatments were shown in Figure 1. There was a considerable number of yeast colonies growing on the plate of 10^–5^ dilution for samples without freeze–thaw treatment (Figure 1a), and yeast used in this study was extremely intolerant to the cold treatment and could barely grow on the PDA plates (Figure 1b). However, the addition of SCMH (Figure 1c) and glycerol (Figure 1d) led to a much better survival rate of yeast undergoing freeze–thaw cycles. After one freeze–thaw cycle, the survival rate of the SCMH‐treated yeast was 90.95%, significantly higher (*p* < .05) than that of both glycerol treated (65.21%) and the control (0.71%); after two freeze–thaw cycles, the survival rate of the SCMH‐treated yeast was still 81.68%, displaying strong protective effect of SCMH on yeast cells against the cold treatment (Figure 1e).

Moreover, broth culture of frozen yeast from the SCMH group (Figure 1g) was opaque after rejuvenation for 24 hr but that from the CTRL (Figure 1f) group seemed still as transparent as fresh medium, indicating that frozen yeast of SCMH group retained good viability in growth and reproduction and further proving that SCMH is an excellent cryoprotectant for yeast.

### 3.2 Effects of SCMH on the ultrastructure of yeast cells

**Figure 2:**
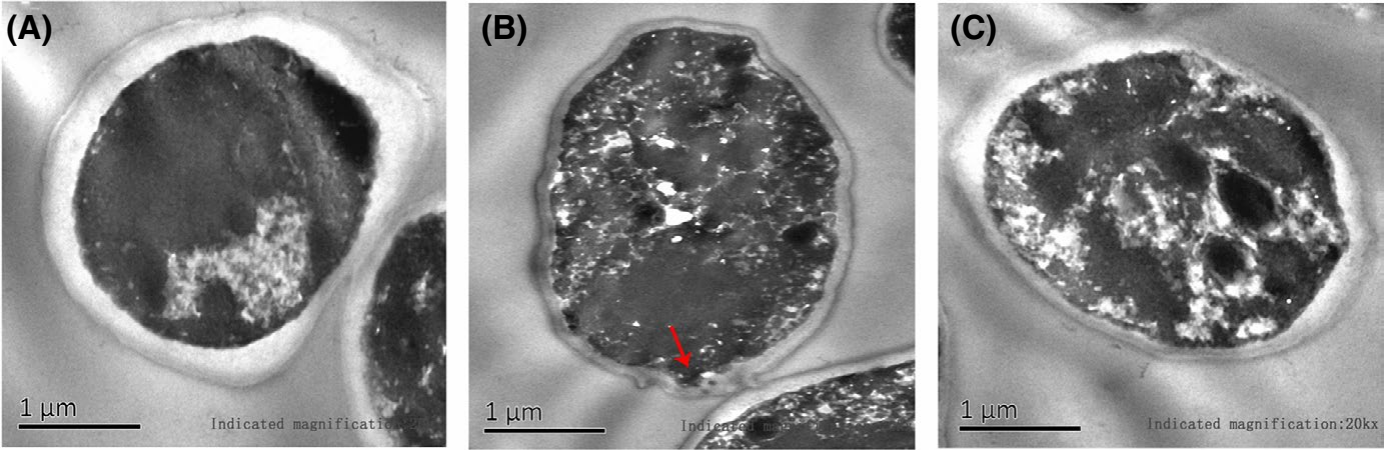
TEM images of the ultrastructure (20,000×) of yeast cells before (A) and after freeze–thaw treatment without SCMH (B) and with 4% of SCMH (C)

An easily recognized cytoskeleton and a typical cell structure can be clearly observed in the unfrozen fresh yeast cell (Figure 2a). After one freeze–thaw cycle, the yeast cell without addition of SCMH showed a thinner and darker cell wall which was partially damaged (pointed by an arrow, Figure 2b), but this corresponding cell treated with 4% of SCMH appeared to be similar to the unfrozen fresh one had a relatively clear, smooth border, and no injury in cell wall (Figure 2c).

### 3.3 Effects of SCMH on proteins and fatty acids in yeast cells

**Figure 3:**
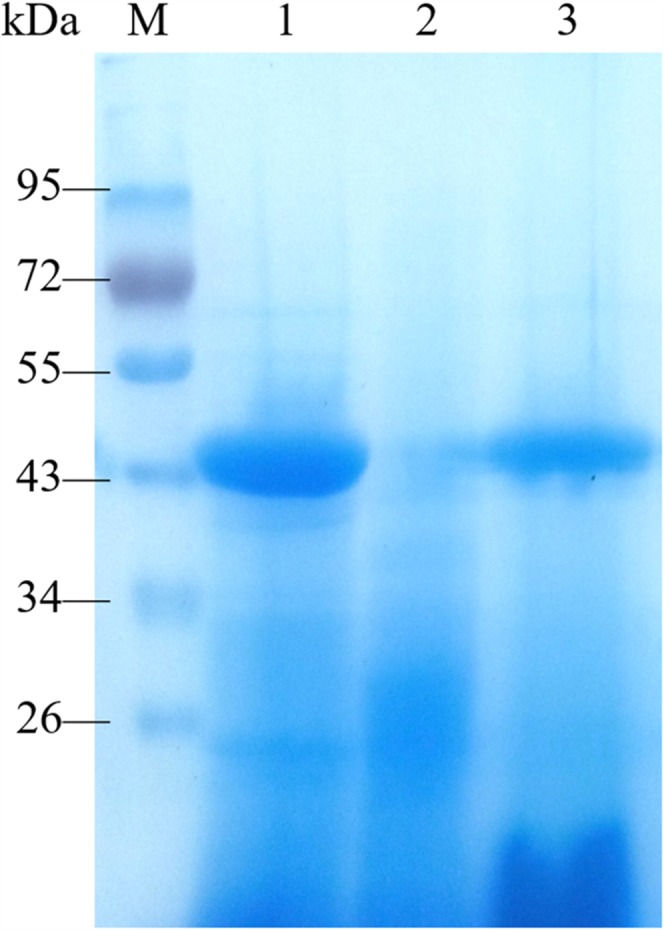
SDS‐PAGE photograph of soluble proteins yeast cells before (Lane 1) and after freeze‐thaw treatment with the incorporation of 0% of SCMH (Lane 2) and 4% of SCMH (Lane 3), M is the low molecular weight marker

The SDS‐PAGE patterns showed that the protein with molecular weight of 45 kDa from frozen yeast cells was almost disappeared (Figure 3, lane 2) after one freeze–thaw cycle. However, the relative staining intensity of the protein band for frozen yeast cells with SCMH was only slightly decreased (Figure 3, lane 3).

**Figure 4:**
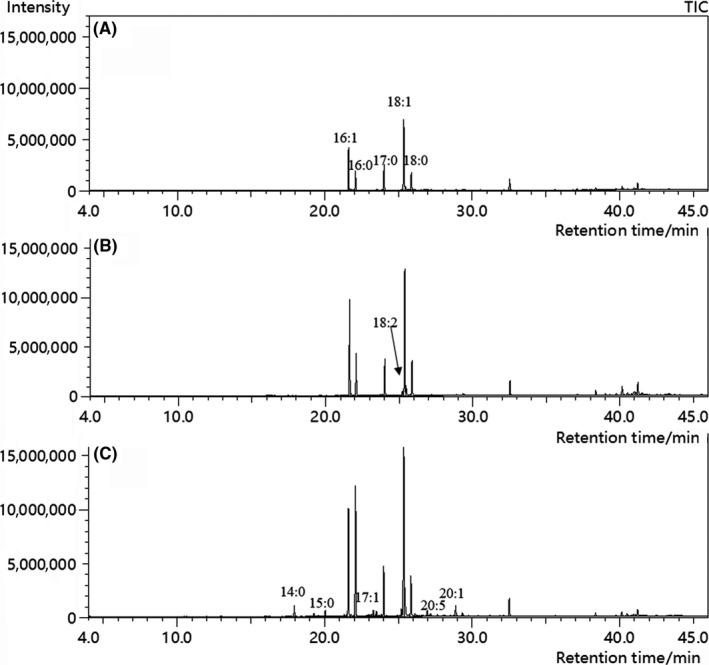
The total ion chromatogram of the fatty acid methyl esters from yeast cells before (A) and after freeze‐thaw treatment with the incorporation of 0% of SCMH (B) and 4% of SCMH (C)

Figure 4 shows the total ion chromatogram of the fatty acid methyl esters from yeast cell samples, and obvious differences in peak intensity or composition could be found between fresh and freeze‐thaw‐treated yeasts, especially for the yeast treated with SCMH (Figure 4a). The major fatty acids found in fresh yeast were palmitoleic (16:1), oleic (18:1), palmitic (16:0), and stearic (18:0) acids, ranging from 9.107 mg/g to 5.879 mg/g on a dry weight basis of yeast cells (Table 1). In addition, heptadecanoic (17:0, 1.820 mg/g), linoleic (18:2, 0.178 mg/g), and myristic (14:0, 0.138 mg/g) acids were detected as minor components, this results were in line with the works of other groups (Bendová, Richter, Janderová, & Häusler, 1991; Murakami, Yokoigawa, Kawai, & Kawai, 1996).

**Table 1:** The amounts of main fatty acids in yeast cells

Fatty acid (mg/g dry weight of cells)	RT time (min)	Fresh yeast	Freeze–thaw‐treated yeast	
			0%SCMH	4% SCMH
Myristic acid (14:0)	17.634	0.138 ± 0.015^b^	0.143 ± 0.014^b^	1.682 ± 0.076^a^
Pentadecoic acid (15:0)	20.051	ND	0.071 ± 0.001^b^	0.141 ± 0.011^a^
Palmitic acid (16:0)	22.071	5.618 ± 0.073^b^	6.589 ± 0.119^b^	21.682 ± 1.370^a^
Palmitoleic acid (16:1)	21.615	9.107 ± 0.375^c^	12.009 ± 0.539^b^	15.986 ± 0.698^a^
Heptadecanoic acid (17:0)	24.005	1.820 ± 0.092^c^	2.584 ± 0.152^b^	5.172 ± 0.155^a^
Stearic acid (18:0)	25.871	5.879 ± 0.298^b^	6.202 ± 0.294^ab^	6.708 ± 0.251^a^
Oleic acid (18:1)	25.354	6.812 ± 0.128^b^	7.200 ± 0.209^b^	11.944 ± 0.392^a^
Linoleic acid (18:2)	25.202	0.178 ± 0.008^c^	0.746 ± 0.021^b^	1.565 ± 0.081^a^
∑SFA	‐	13.509 ± 0.664^c^	15.694 ± 0.859^b^	35.605 ± 1.327^a^
∑UFA	‐	16.407 ± 0.922^c^	20.674 ± 1.445^b^	30.392 ± 0.898^a^

Note

Data were presented as mean ± (*SD*); values within the same row bearing different superscript letters (a, b, and c) are significantly different (*p* < .05). ∑SFA and ∑UFA represent the total content of saturated fatty acids and unsaturated fatty acids, respectively；ND is no detected.

As shown in Table 1, there has been a substantial increase in absolute content of all identified fatty acids in yeast cells (on dry weight basis) after a freeze‐thaw treatment, but the increasing extent of almost all fatty acids for the yeast cells without SCMH treatment was significantly less than (*p* < .05) that of yeast cells with 4% of SCMH treatment. As a consequence, the total content of fatty acids in yeast cells were significantly (*p* < .05) increased after a freeze‐thaw treatment; the amount of saturated and unsaturated fatty acids in the yeast cells with 4% of SCMH was increased to 35.605 and 30.392 mg/g as compared to that of 13.509 and 16.407 mg/g in fresh group without freeze–thaw treatment, respectively, significantly higher (*p* < .05) than that of the group without SCMH (the two corresponding values were 15.694 and 20.674 mg/g, respectively) (Table 1). In addition, some fatty acids detected in freeze‐thaw‐treated yeast were not found in untreated yeast cells. For examples, the pentadecoic (15:0) and omega‐3 eicosapentaenoic (EPA, 20:5) acids were detected in both freeze–thaw‐treated samples (Figure 4b and c), and the cis‐10‐heptadecenoic (17:1), tricosanic (23:0), and eicosenoic (20:1) acids were detected only in freeze–thaw‐treated samples with addition of SCMH, but none of these fatty acids were found in untreated yeast cells (Figure 4c).

### 3.4 Effects of SCMH on external and internal ice formation

**Figure 5:**
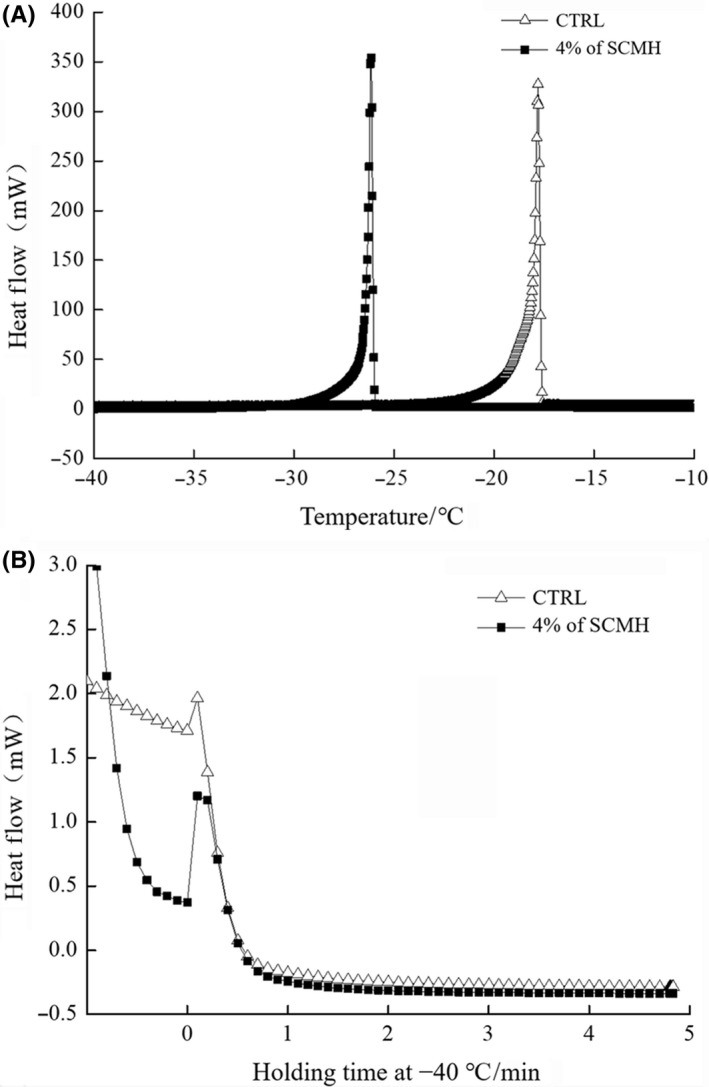
The change of external ice formation (EIF) (A) and intracellular ice formation (IIF) (B) peak of frozen yeast suspension with the incorporation of SCMH determined by DSC

Figure 5 shows the DSC exotherm characteristic of frozen yeast suspensions with or without the addition of SCMH, and a large peak occurred at continuous cooling process and a very small peak was observed when holding at −40°C. As Seki, Kleinhans, and Mazur (2009) suggested, the large peak represented the external ice formation (EIF) peak and the small peak represented internal ice formation (IIF) peak. So, our results corroborated Jia's results (2012) that IIF was formed in yeast suspensions.

An obvious drop in the EIF peak temperature was observed with the addition of SCMH, shifting from –17.95°C to –25.14°C (Figure 5a). This hysteresis effect suggested that SCMH could decrease the freezing point of biological fluids and delay their eutectic crystallization process. Moreover, compared with the CTRL group, the exothermic peak of SCMH group due to IIF was distinctly decreased (Figure 5b), indicating much less intracellular ice formation in yeast cells with the addition of SCMH.

## 4 DISCUSSION

It is evidenced that the dying of yeast undergoing freezing stress is related to the quality deterioration of frozen dough (Autio & Mattila‐Sandholm, 1992; Wolt & Appolonia, 1984). Our results showed that the SCMH exhibited excellent yeast cryoprotective effect, leading to 90.95% survival rate of yeast cells undergoing one freeze–thaw cycle as compared to that of 0.71% for the control without SCMH added (Figure 1), indicating that SCMH is a favorable cryoprotectant for baker's yeast.

In order to investigate the effect of SCMH on yeast cells, the ultrastructure of freeze–thaw‐treated cells with or without addition of SCMH was firstly probed using TEM technique. The damage on cell membrane and cell wall of yeast cells could be clearly observed after free‐thaw treatment without SCMH, which is similar to that reported by Mazur (1984). The addition of 4% SCMH attenuated the freeze damage of yeast cells, which may explain the significant increase of yeast survival rate.

Murakami et al. (1996) reported that the susceptibility of yeast cells to freeze injury may be partly associated with biochemical features of the protein composition and membrane lipids in yeast. Therefore, the difference of soluble proteins and fatty acid profiles of fresh and frozen yeast was compared using SDS‐PAGE and GC‐MS (Figure 4), respectively. The SDS‐PAGE profile in Figure 3 confirmed the previous results that freezing could cause not only damage to the cell wall and membrane but also denaturation of functional proteins (Attfield, 1997; Shima & Takagi, 2009); Leslie, Israeli, Lighthart, Crowe, and Crowe (1995) also reported that protein structure in intact bacteria upon freezing stress had changed and trehalose or sucrose could prevent this change. So our results imply that the addition of SCMH could greatly alleviate the degradation or loss of yeast intracellular proteins during freeze–thaw cycle, playing a cryoprotective role in preventing serious injury in yeast cell structure. Moreover, previous studies (Redón, Guillamón, Mas, & Rozès, 2011; Suutari, Liukkonen, & Laakso, 1990) have shown that higher levels of unsaturated fatty acids in yeast cells helped increase the membrane fluidity and resulted in superior temperature adaptation for less freeze injury. Our results (Table 1) were consistent with the reported data (Annous, Becker, & Bayle, Labeda, & Wilkinson, 1997; Murakami et al., 1996) and indicated that a higher amount of fatty acids were likely expressed in yeast cells at low‐temperature so as to reduce freezing injury by increasing the fluidity of plasma membrane. The addition of SCMH seemed to be conducive to this induction mechanism, thereby enhanced freeze tolerance of yeast.

Furthermore, the yeast cells incorporation with 4% of SCMH caused a delay in the EIF and a decrease in the IIF (Figure 5). As several other investigators (Jia et al., 2012; Seki et al., 2009) suggested, this is probably because that the addition of SCMH led to longer time for yeast cells to dehydrate by lagging EIF process, resulting in the decrease in IIF and less freezing damage. In an effort to further reveal why IIF was decreased, DSC was also employed to determine the freezable water content in yeast cells. The results showed that freezable water content in CTRL yeast cells was 66.82 ± 3.02%, while it was down to 60.30 ± 2.13% when 4% of SCMH was added. The significant (*p* < .05) decrease in freezable water content may interpret IIF decline of yeast with the addition of SCMH. Kuiper, Lankin, Gauthier, Walker, and Davies (2003) and Zhang et al. (2008) reported that AFPs can absorb free water around them with the consequence to restrict a free movement of water. Therefore, the addition of SCMH may also lead to an increase in the proportion of nonfreezable water in yeast cells following similar mechanism, thus reducing the freezable water content in yeast cells inhibiting the IIF and attenuating its freeze damage to cells.

## 5 CONCLUSIONS

Our results showed that the SCMH exhibited excellent yeast cryoprotective effect, leading to 90.95% survival rate of yeast undergoing one freeze–thaw cycle as compared to that of 0.71% for the control without SCMH added. Two possible underlying mechanisms for the protecting effect of SCMH on yeast in cold environment are proposed here. Firstly, the addition of SCMH could reduce or slow down the degradation or loss of intracellular proteins and increase expression of fatty acids in yeast cells, thus enhance the fluidity of cell membrane and attenuate the freeze damage of yeast cellular structure. Secondly, the addition of SCMH could delay the yeast EIF process and increase the proportion of nonfreezable water, alleviating the IIF and attenuating its freeze damage to yeast cells.

## CONFLICT OF INTEREST

The authors declare that they have no conflict of interest.

## ETHICAL APPROVAL

This study does not involve any human testing. All applicable international, national, and/or institutional guidelines for the care and use of animals were followed.
